# Psychometric properties of the Disability of Arm Shoulder and Hand (DASH) in subjects with frozen shoulder: a reliability and validity study

**DOI:** 10.1186/s12891-024-07371-8

**Published:** 2024-04-02

**Authors:** Fabrizio Brindisino, Davide Venturin, Matteo Bartoli, Serena Caselli, Leonardo Pellicciari, Antonio Poser

**Affiliations:** 1 Department of Medicine and Health Science “Vincenzo Tiberio”, University of Molise c/o Cardarelli Hospital, C/da Tappino, Campobasso, 86100 Italy; 2 Physiotherapy private practice KinesiLab, via Marcantonio Colonna 88, Marino, Italy; 3 Unità Operativa di Medicina Riabilitativa, Azienda Ospedaliero-Universitaria di Modena, Modena, Italy; 4 IRCCS Istituto delle Scienze Neurologiche di Bologna, Via Altura, 3, Bologna, I-40139 Italy; 5 Physiotherapy private practice Kinè, Kinè s.r.l, Viale della Quercia 2/B,, Treviso, Italy; 6 University of Siena, c/o via Banchi di Sotto, 55, Siena, Italy

**Keywords:** Psychometrics, Reproducibility of results, Validation studies, Patient-reported outcome measures, Rehabilitation, Adhesive capsulitis

## Abstract

**Background:**

Frozen Shoulder (FS) is a painful condition characterized by severe pain and progressive restriction of shoulder movement, leading to functional impairment and reduced quality of life. While different Patient Reported Outcome Measurements (PROMs) tools exist for assessing shoulder diseases, few specific PROMs are validated for FS patients.

**Purpose:**

This study aims to assess the psychometric properties of the Disability of Arm, Shoulder, and Hand (DASH) questionnaire in FS patients.

**Methods:**

One hundred and twenty-four subjects (mean ± SD age = 55.4 ± 7.9 years; 55.6% female) diagnosed with FS were included and completed the DASH questionnaire, the Numerical Pain Rating Scale (NPRS), the Shoulder Pain and Disability Index (SPADI), and the Short-Form Health Survey 36 (SF-36). Floor or ceiling effects were investigated. Structural validity was analysed through a unidimensional Confirmatory Factor Analysis (CFA), internal consistency through Cronbach’s alpha, test-retest reliability through the Intraclass Correlation coefficient (ICC), measurement error through the Standard Error of Measurement (SEM), and the Minimum Detectable Change (MDC), and construct validity through the hypothesis testing with the correlation with the other outcome measures used.

**Results:**

No floor or ceiling effects were observed. CFA confirmed a one-factor structure after addressing local item dependency (Root Mean Square Error of Approximation = 0.055; Standardized Root Mean Square Residual = 0.077; Comparative Fit Index = 0.970; Tucker-Lewis Index = 0.968). Cronbach’s alpha was high (= 0.951), and test-retest reliability was excellent (ICC = 0.999; 95% CI: 0.998-1.000). SEM was equal to 0.5 points, and MDC to 1.5 points. Construct validity was considered satisfactory as 80% of the *a-priori* hypotheses were met.

**Conclusion:**

The DASH questionnaire demonstrated good psychometric properties in FS patients, supporting its use as a valuable tool for assessing the impact of FS in clinical and research settings.

## Background

Frozen Shoulder (FS) is a debilitating condition affecting the shoulder joint characterized by the progressive and painful restriction of active and passive movement [1], leading to considerable functional impairment and reduced quality of life [2]. Clinically, FS causes an insidious and worsening daily and nightly pain, severe restriction of shoulder range of motion (ROM), and disability in the absence of significant radiographic findings [3, 4], and is often associated with sleep deprivation, depression, anxiety, and fear avoidance beliefs [2, 5, 6].

The evidence in the literature for the “self-limiting” and “3-phase” theory appears to be debatable. At the same time, it is suggested that there is an early improvement in disabilities (with the greatest gain in the early disease process) that slows with time [7]. Consequently, prolonged limitations in active and passive ROM and functionality can last long, with no evidence for complete recovery without supervised treatment [8]. Moreover, patients with severe complaints, diabetes, more movement restriction, and more co-morbidities have a worse prognosis for recovery [9] with some subjects that could complaint a slight painful and restricted shoulder (in terms of mobility and functionality) after a certain treatment period [10].

Prevalence of FS is estimated at 2–4% of the general population; gender predisposition is a matter of debate, and subjects of working age (particularly between 40 and 60 years) are most commonly affected [11]. Several factors are associated with FS, such as trauma, diabetes, thyroid and autoimmune pathologies, familiar history [12–14], and Dupuytren disease [15]. Diagnosis is mainly clinical [16], and management usually focuses on pain reduction and restoration of ROM and function. In particular, treatments such as corticosteroid injections [17], patient education [2], stretching [18], exercise therapy [19, 20], and joint mobilization [21] are recommended, while the use of electrophysical agents was not supported [22].

Since FS is a long-lasting condition [23–25] and implies a struggle to maintain a normal life while lining up with the significant pain, physical restriction, sleep loss, and disability experienced [26], it is important to determine as better as possible the impact of FS in subjects’ lives to evaluate outcomes. The availability of a valid Patient Reported Outcome Measure (PROM) is recommended in clinical practice and research [27]. PROMs were considered the best instruments to assess health-related domains [28], as they capture the subject’s perspective of the impact of disease on the individual [29]. To the authors’ knowledge, no current specific PROMs are specially developed for FS patients. However, some PROMs were validated, and psychometric properties were investigated, particularly for subjects affected by FS, such as the Shoulder Pain and Disability Index (SPADI) [30] and Patient-Reported Outcomes Measurement Information System (PROMIS) [31]. Moreover, the American Shoulder and Elbow Surgeon score (ASES) [32], and the Upper Limb Functional Index (ULFI) [33] were validated in samples of patients suffering from musculoskeletal shoulder pathologies, including FS.

The Disability of Arm, Shoulder, and Hand (DASH) [34] questionnaire is one of several PROMs in the literature for assessing outcomes in subjects with shoulder diseases [34, 35]. It was designed to help describe the disability experienced and its impact on daily life in several upper limb activities. DASH showed good psychometric values in many shoulder pathologies [35–38]. The use of the DASH [34] score is useful in assessing upper limb functional limitation, and it is highly recommended before and following interventions aimed at describing impairments of body functions and structures, limitation in activities, and restriction in participation (according to the International Classification of Functioning, Disability and Health) linked to FS [16].

DASH was widely translated and used in literature [39–42], but its psychometric properties in subjects affected by FS are currently lacking. Only the Persian version DASH study [36] enrolled a sample with different limb musculoskeletal pathologies, including FS patients.

As the psychometric properties of an instrument are influenced by social, environmental, and clinical factors specific to a cluster of subjects suffering from a particular disease [43], it is necessary to assess the DASH psychometric properties in subjects with FS to use it in this population properly.

For these reasons, this study aims to assess the DASH psychometric properties (i.e., reliability and validity) in a sample of subjects suffering from FS.

## Methods

### Participants

Participants were concurrently recruited from physiotherapy private practices in Lecce, Conegliano Veneto, and Latina (Italy) from April to October, 2023. The inclusion criteria consisted of subjects over 18 years old with a clinical diagnosis of idiopathic FS, according to Kelley et al., 2013 guidelines [16]. In particular, subjects had complained of insidious onset of shoulder pain and limitation of active and passive ROM by exceeding 50% loss in external rotation at arm by side when compared to the unaffected side and a minimum of 25% ROM loss in at least two planes of movement. In addition, symptoms had to be stable for at least one month or worsen with a negative radiological evaluation [16].

In each center, a physiotherapist with a high experience in shoulder complaints screened the subjects following the above-mentioned criteria. Moreover, to ensure the correct diagnosis, subjects were referred to an orthopedic with expertise in shoulder pathologies for further consultation: in all cases, both professionals agreed on the diagnosis of FS.

We excluded subjects with post-traumatic and/or postoperative capsulitis and those who were not Italian-speaking or presented cognitive impairments.

This study was approved by the Ethics Committee of the University of Molise (protocol number 22/2023). All subjects signed informed consent.

### Outcome measures

The *DASH* questionnaire [34] consists of 30 items investigating the difficulty in performing several daily activities using the upper limb, pain, stiffness, weakness, tingling, and impact on social activities, sleep, and work [44]. Patients are asked to assess their level of difficulty for each question using a five-point Likert scale, which spans from “no difficulty or no symptoms” (i.e., scoring 1 point) to “unable to perform the activity or experiencing severe symptoms” (i.e., scoring 5 points) [45]. All these items compose the total score computed by summing every subscale score. Then, the total score is converted to a scale from 0 to 100 points, where 0 indicates no disability, and 100 represents the most severe disability. The Italian version of the DASH [38] was used in this study.

The *Numerical Pain Rating Scale* (NPRS) is an eleven-point scale that rates pain intensity in subjects by assigning a numeric value from 0 (marked as “no pain”) to 10 (marked as “worst possible pain”) points [46]. This outcome measure was widely used in research and has proven valid and reliable in assessing shoulder pain [47]. Still, it has not yet been used to determine pain intensity in FS subjects [30, 48].

The *Shoulder Pain and Disability Index* (SPADI) [49] is a self-report questionnaire used to assess the severity of shoulder pain and its impact on daily activities and functional limitations. The questionnaire typically consists of 13 items divided into a pain subscale (5 items) and a disability subscale (8 items) that assess the degree of pain and difficulty experienced by the individual when performing various shoulder-related tasks, such as reaching, lifting, and sleeping. For each item, the patient must assign a score ranging from 0 to 10 points, where a higher score indicates a greater level of pain or disability. The total score of the SPADI is calculated by averaging the results of the two subscales. In each subscale, the patient can choose to skip a response, and that item will also be excluded from the total score calculation; however, if the patient omits a response to more than two items, it will not be possible to calculate the total score. This study used the Italian version of SPADI [50] since it showed robust psychometric properties in subjects with FS [30].

The *Short Form Health Survey 36* (SF-36) [51] is a widely used generic health-related quality of life questionnaire. It is designed to assess a person’s perception of his/her health and well-being across various dimensions. Moreover, it can be applied to multiple health conditions and populations, making it a versatile tool for evaluating health-related quality-of-life outcomes. The SF-36 consists of 36 questions that are divided into eight health domains: Physical Functioning (PF; 10 items), Role Limitations due to Physical Health Problems (RP; 4 items), Role Limitations due to Emotional Health Problems (RE; 3 items), Vitality (VT; 4 items), Mental Health (MH; 5 items), Social Functioning (SF; 2 items), Bodily Pain (BP; 2 items) and General Health Perception (GH; 5 items). Each of these dimensions is assessed through several questions, and the responses are scored and weighted to calculate scores for each domain. These domain scores can then be aggregated to provide two summary scores: the Physical Component Summary (PCS) score, which is formed by BP, PF, GH, and RP subscales, and the Mental Component Summary (MCS) score, composed of MH, VT, SF, and RE subscales. The responses to questions within each score are combined and transformed into a scale ranging from 0 to 100 points. Data are then normalized and standardized to facilitate comparisons across different populations. The transformation involves converting the raw scores to a scale where higher values represent better health-related quality of life. The Italian version of the SF-36[52] was used in this study.

### Procedures

The DASH, NPRS, SPADI, and SF-36 were administered to each subject. In addition, an ad hoc questionnaire was administered to collect demographic (i.e., age, gender, occupation, dominant arm) and clinical (i.e., symptom onset, shoulder affected, comorbidities) data. The DASH was asked to be filled in after five days from the first administration to a sub-sample to assess the test-retest reliability; between the two administrations of DASH, no treatment was provided to keep the subjects’ clinical condition stable.

### Statistical analysis

Psychometric properties of the DASH questionnaire were investigated following COnsensus-based standards for the Selection of health Measurement INstruments (COSMIN) initiative recommendations [53].

#### Sample characteristics

The sample descriptive statistics comprised the mean ± standard deviation (SD) for interval variables, the median with the first and third quartile for ordinal variables, and the frequency with percentage for categorical variables.

#### Acceptability

Acceptability was measured as the existence of multiple and/or absent answers [54]. To gauge potential floor and ceiling effects, it was considered noteworthy when more than 15% of the patients obtained the lowest or highest score [55], respectively.

#### Structural validity

A Confirmatory Factor Analysis (CFA) was run to assess the structural validity of the DASH questionnaire. Data were submitted to a one-factor model to confirm unidimensionality, obtaining a single unidimensional questionnaire for clinical use. Within the context of the CFA, we considered the following indicators as a good fit to the model: the Root Mean Square Error of Approximation (RMSEA) ≤ 0.06[56], the Standardized Root Mean Square Residual (SRMR) ≤ 0.08[56], the Comparative Fit Index (CFI) and the Tucker-Lewis Index (TLI) ≥ 0.95[56]. Moreover, the factor loading for each item was reported; a factor loading higher than 0.40 was considered acceptable [57]. If the baseline analysis failed to fit the one-factor model, the local dependence (i.e., the response to one of the items is influenced by the response of another item [58]) between item pairs was assessed by examining the modification indices (MI) [59], indicators of model misspecification. If the local dependence was detected, the model was re-specified by allowing correlation of the error terms of the items in the pair. Following each modification, the model fit was reassessed again. This analysis strategy was used until no other modification was possible.

#### Reliability

In terms of reliability, internal consistency, test-retest reliability, and measurement error were assessed. Internal consistency was evaluated utilizing the following indexes: (1) Cronbach’s alpha (α), with suggested values falling over 0.90 for individual measurement [59]; (2) the average inter-item correlations, assessed by the Spearman’s correlation coefficient [60], with acceptable values ≥ 0.200[61]; (3) item-to-total correlation (ITC), as determined by Spearman’s correlations (r_s_) where values ≥ 0.25 were deemed acceptable [62] and (4) α if an item was deleted, with expected values lower than the total α [63].

Test-retest reliability was established by computing the Intraclass Correlation Coefficient (ICC_2,1_) using a 2-way random effects model with a 95% confidence interval (CI). Aimed to lower the selection bias, the test-retest sample was a sub-sample with consecutive recruitment.

Reliability was affirmed by ICC values exceeding 0.75 for group-level measurements and 0.85 for individual-level measurements [64]. Moreover, ICC was considered poor if the value was below 0.50, moderate if the value was between 0.50 and 0.75, good if the value was between 0.75 and 0.90, and excellent if the value was above 0.90[65].

Measurement error was assessed using the Standard Error of Measurement (SEM) and the Minimum Detectable Change (MDC). SEM was computed using the formula SD*√ (1 – ICC), with SD being the baseline measurements’ SD and the ICC value derived from the test-retest reliability. MDC was calculated by multiplying the SEM by 1.96, corresponding to the z-score associated with a 95% confidence level and the square root of 2[57].

#### Construct validity

In terms of validity, construct validity was also investigated through *a-priori* hypotheses testing using Spearman’s correlations rank (r_s_) between DASH questionnaire and the other questionnaires subscales administrated (i.e., SPADI pain and disability subscales, NPRS, and PCS and MCS scores for SF-36). Spearman’s rank correlation cut-offs were considered strong if r_s_ was ≥ 0.60, moderate if r_s_ was between ≥ 0.30 and < 0.60, and weak if r_s_ was < 0.30[66]. The a-priori hypotheses were the following:

1. The correlation between DASH and NPRS was presumed to be moderate (i.e., r_s_ ≥ 0.30 and < 0.60) because pain (measured as pressure pain threshold) has shown to have a strong to fair connection with disability in subjects with FS [7, 67];
2. The correlation between DASH and SPADI Pain subscale was thought to be strong (i.e., r_s_ ≥ 0.60) because the pain has shown a strong to fair connection with disability in subjects with painful shoulders. This hypothesis is different from the previous hypothesis because, unlike the NRS, the SPADI Pain subscale measures pain from the point of view of disability, a variable that the DASH assesses;
3. The correlation between DASH and SPADI Disability subscale score was expected to be strong (i.e., r_s_ ≥ 0.60) because both assess the impact of pathology on activities and participation and assess similar aspects (i.e., disability);
4. The inverse correlation between DASH and SF-36 MCS was presumed to be strong (i.e., r_s_ ≥ -0.60) because there is evidence about connections between motor function and psychological distress in patients with FS [5];
5. The inverse correlation between DASH and SF-36 PCS was presumed to be moderate (r_s_ ≥ -0.30 and < -0.60) because the instruments measure interconnected variables, yet not identical.

This construct validity was evaluated as satisfactory, mild, and insufficient if values ≥ 75%, between ≥ 50% and < 75%, and < 50% of the *a-priori* hypotheses were satisfied, respectively [54].

#### Sample size and software issues

As COSMIN initiative suggests [68], a minimum of 100 subjects for studying structural validity through factor analysis were needed.

CFA was conducted using Mplus software (version 6.0, Muthen & Muthen, Los Angeles, CA; 1998–2010; www.statmodel.com), while other statistics were computed by SPSS software (version 21 for Windows; SPSS Inc., Chicago, IL; 2004).

## Results

### Sample characteristics

One hundred twenty-four subjects (mean ± SD age = 55.4 ± 7.9 years; 55.6% female) agreed to participate to the study. The mean ± SD onset of FS was 6.9 ± 4.7 months, and data showed that in most recruited subjects, the non-dominant shoulder was affected (54.8%). Detailed demographic and clinical characteristics of the sample are shown in Table 1.

**Table 1:** Demographic and clinical characteristics of the sample (*N* = 124)

Variable	Mean ± SD	Frequency (%)	Median (1st, 3rd quartile)
Age (*years)*	55.4 ± 7.9		
Gender			
Female		69 (55.6%)	
Male		55 (44.4%)	
Onset, *months*	6.9 ± 4.7		
Shoulder affected			
Left		68 (54.8%)	
Right		56 (45.2%)	
Dominant arm			
Right		112 (90.3%)	
Left		12 (9.7%)	
Occupation			
Employed		66 (53.2%)	
Self-employed		39 (31.5%)	
Retired		14 (11.3%)	
Housewife		5 (4.0%)	
Comorbidities			
Diabetes		13 (10.5%)	
Hypertension		9 (7.3%)	
Thyroid pathologies		7 (5.6%)	
Hearth diseases		3 (2.4%)	
Autoimmune diseases		2 (1.6%)	
Rheumatological pathologies		2 (1.6%)	
Epilepsy		2 (1.6%)	
Migraine		1 (0.8%)	
Chronic gastritis		1 (0.8%)	
Hypercholesterolemia		1 (0.8%)	
Hyperglycemia		1 (0.8%)	
Prostatic hypertrophy		1 (0.8%)	
Pulmonary tumor		1 (0.8%)	
Osteoporosis		1 (0.8%)	
Extrapyramidal syndrome		1 (0.8%)	
Hormone therapy		1 (0.8%)	
Leukocytoclastic vasculitis		1 (0.8%)	
None		76 (61.3%)	
DASH			39.5 (25.0, 52.0)
NPRS			7.0 (5.0, 8.0)
SPADI			
Pain subscale			59.0 (38.5, 76.0)
Disability subscale			50.5 (30.0, 70.0)
SF-36			
PCS			41.2 (36.6, 46.0)
MCS			40.1 (22.3, 50.5)

**Abbreviations:** SD, Standard Deviation; %, percentage; DASH, Disability of the Arm, Shoulder and Hand; NPRS, Numeric Pain Rating Scale; SPADI, Shoulder Pain and Disability Index; SF-36, Short Form Health Survey 36; PCS, Physical Component Summary; MCS, Mental Component Summary.

### Acceptability

All individuals in the sample responded to every item except for item#21, where responses from six (6/124 = 0.05%) participants were missed. The absence of any floor or ceiling effects resulted.

### Structural *validity*

Regarding structural validity, the base analysis showed a misfit to a one-factor model (RMSEA = 0.084; SRMR = 0.089; CFI = 0.930; TLI = 0.925). After accounting for local dependence between several item pairs, data showed a proper fit to the model (RMSEA = 0.055; SRMR = 0.077; CFI = 0.970; TLI = 0.968). Factor loading of the final analysis for each item was ≥ 0.400 (Table 2).

**Table 2:** Item descriptive statistics, confirmatory factor analysis, and internal consistency results of the Disability, aAm, Shoulder and Hand questionnaire (*N* = 124)

Item labels	Descriptive statistics		Confirmatory factor analysis						Internal consistency	
			*Baseline analysis*			*Final analysis*				
	*Mean ± SD*		*Factor Loading*	*SE*		*Factor Loading*	*SE*		*ITC*	*αIID*
1. Open a tight or new jar	2.8 ± 1.3		0.711	0.045		0.724	0.046		0.677	0.949
2. Write	1.3 ± 0.6		0.529	0.092		0.538	0.094		**0.368**	**0.951**
3. Turn a key	1.6 ± 0.9		0.600	0.071		0.568	0.075		0.473	0.950
4. Prepare a meal	1.7 ± 0.8		0.722	0.050		0.703	0.054		0.621	0.949
5. Push open a heavy door	2.7 ± 1.1		0.736	0.043		0.751	0.043		0.679	0.948
6. Place object on shelf above your head	3.5 ± 1.1		0.812	0.033		0.822	0.033		0.745	0.948
7. Do heavy household chores	3.0 ± 1.1		0.801	0.035		0.815	0.035		0.739	0.948
8. Garden or do yard work	2.8 ± 1.1		0.687	0.046		0.700	0.047		0.643	0.949
9. Make a bed	2.5 ± 1.1		0.822	0.032		0.837	0.032		0.759	0.948
10. Carry a shopping bag or briefcase	2.4 ± 1.1		0.785	0.033		0.720	0.040		0.670	0.949
11. Carry a heavy object (over 10 lbs)	2.8 ± 1.1		0.796	0.034		0.728	0.042		0.693	0.948
12. Change a lightbulb overhead	3.6 ± 1.2		0.820	0.031		0.831	0.031		0.747	0.948
13. Wash or blow dry your hair	2.8 ± 1.3		0.704	0.045		0.715	0.045		0.677	0.949
14. Wash your back	3.9 ± 1.1		0.719	0.046		0.733	0.047		0.653	0.949
15. Put on a pullover sweater	3.0 ± 1.1		0.678	0.047		0.688	0.047		0.621	0.949
16. Use a knife to cut food	1.8 ± 1.0		0.654	0.052		0.667	0.053		0.573	0.949
17. Recreational: little effort	1.6 ± 0.9		0.685	0.055		0.696	0.056		0.533	0.950
18. Recreational: force	3.3 ± 1.2		0.686	0.045		0.644	0.052		0.641	0.949
19. Recreational: free arm movement	3.5 ± 1.1		0.715	0.049		0.679	0.054		0.662	0.949
20. Manage transportation needs	1.4 ± 0.8		**0.393**	0.084		0.400	0.085		**0.287**	**0.951**
21. Sexual activities	1.5 ± 0.5		0.438	0.076		0.447	0.077		**0.367**	**0.951**
22. Interference with social activities	2.7 ± 1.0		0.714	0.042		0.635	0.054		0.616	0.949
23. Limitation in work and daily activities	2.6 ± 1.0		0.744	0.037		0.697	0.043		0.678	0.949
24. Arm, shoulder or hand pain	2.9 ± 0.9		0.712	0.045		0.644	0.053		0.643	0.949
25. Pain during activity	2.9 ± 0.8		0.704	0.053		0.686	0.055		0.762	0.948
26. Tingling	1.9 ± 1.0		0.530	0.063		0.540	0.064		0.524	0.950
27. Weakness	2.5 ± 1.0		0.594	0.056		0.607	0.057		0.568	0.949
28. Stiffness	3.1 ± 1.1		0.498	0.059		0.509	0.060		0.463	**0.951**
29. Sleep difficulty	2.8 ± 1.2		0.699	0.051		0.662	0.057		0.650	0.949
30. Capability and confidence	3.1 ± 1.3		0.512	0.066		0.484	0.071		0.505	0.950
*Recommended values*	*N/A*		*≥ 0.400*	*N/A*		*≥ 0.400*	*N/A*		*≥ 0.250*	*<α*

**Abbreviations:** SD, standard deviation; SE, standard error; ITC, Item-to-total correlation; αIID, Cronbach’s α if item deleted; N/A, not applicable

**Notes:** statistics values beyond the recommended cut-off are in bold.

### Reliability

Findings showed a high internal consistency (α = 0.951) for the DASH total score; similar results were obtained for the average inter-item correlations, equal to 0.389. ITC exhibits good values for all DASH items, except for item#2, item#20, and item#21 (ITC = 0.368, 0.287,0.367, respectively) (Table 2). The evaluation of Cronbach’s alpha if an item was deleted showed that DASH’s internal consistency is solid. Indeed, removing most items may not significantly impact the scale’s overall reliability, apart from item#2, item#20, item#21, and item#28 (Table 2). Test-retest reliability, assessed in a subsample of 35 subjects, showed an ICC value of 0.999 (95% CI: 0.998-1.000), demonstrating excellent test-retest reliability. Measurement error findings showed a SEM of 0.5 points (equal to 0.5%) and a MDC of 1.5 points (equivalent to 1.5%).

### Construct validity

Concerning hypothesis-testing, Spearman’s rank analysis confirmed that four out of five (80.0%) a-priori hypotheses were met, so construct validity can be considered satisfactory. Correlation results are shown in Table 3.

**Table 3:** Hypothesis testing for Spearman’s rank correlations between the Disability of Arm, Shoulder, and Hand questionnaire and comparator instruments (*N* = 124)

Hypothesis testing	Estimated correlation	Hypothesis met?
1. The correlation between DASH and NPRS is moderate (i.e., r_s_0.30 ≤ r_s_ < 0.60)	0.484*	Yes
2. The correlation between DASH and SPADI Pain subscale is strong (i.e., r_s_ ≥ 0.60)	0.752*	Yes
3. The correlation between DASH and SPADI Disability subscale is strong (i.e., r_s_ ≥ 0.60)	0.830*	Yes
4. The inverse correlation between DASH and SF-36 MCS is strong (i.e., r_s_ ≥ -0.60)	-0.401*	No
5. The inverse correlation between DASH and SF-36 PCS is moderate (i.e., -0.30 ≤ r_s_ < -0.60)	-0.517*	Yes

**Abbreviations**: DASH, Disability of Arm, Shoulder and Hand; NPRS, Numerical Pain Rating Scale; SPADI, Shoulder Pain and Disability Index; SF-36, Short Form Health Survey 36; PCS, Physical Composite Score, MCS, Mental Composite Score, (r_s_): Spearman’s correlations rank

**Notes:** The numbers of hypotheses refer to the ones reported in the Methods section

* *p* < 0.001.

## Discussion

In the present study, the DASH questionnaire demonstrated good acceptability, proving to be very understandable by the sample, adequate structural validity showing unidimensionality after solving the local dependence, high internal consistency, excellent test-retest reliability, and low values of measurement errors, and satisfactory hypothesis-testing construct validity.

Acceptability was measured considering the way the sample engaged the items. Notably, all individuals in the sample responded to every item except for item#21 (six answers missing), demonstrating a high level of participation and cooperation and proving the scale comprehensible. Moreover, no floor or ceiling effects resulted, showing that the scale could detect changes in disability when it is very low or very high.

The baseline CFA analysis revealed a lack of fit with the unidimensional model. However, following the adjustment for local dependence among item pairs, the data demonstrated a sufficient fit to the model. Additionally, the factor loadings for each item exceeded the threshold of ≥ 0.400, as recommended, underscoring the robustness of the relationships between the latent construct and the observed variables. This sequence of analyses offers valuable insights into the model’s suitability and the data’s fidelity. This suggests that each item contributes meaningfully to the overall construct, reinforcing the validity of the measurement instrument used in the study. To our knowledge, there is a lack of consensus about the number of factors by which the DASH scale is composed [69], leaving authors with variable options for its adaptation. We knew that DASH was widely translated and cross-culturally adapted in many countries, considering it variably as a one-, two-, or three-factor instrument [69–73] in different clinical sample. Particularly, the Rasch analysis studies from Franchignoni et al. [74] and Lehman et al. [75] found three DASH factors, while Basakci et al. [76] found two DASH factors, concluding that the DASH is not unidimensional. However, Wang et al. [37] showed that one main factor explains 62% of the variance, stating that the structure of DASH can be considered as unidimensional, as confirmed also by the Rasch analysis by Van Lieshout et al. [77] Hence, in literature, no consensus is available regarding the DASH factorial structure, as this psychometric property depends either on the clinical variables of the population studied either on the setting in which this instrument is administered.

In this study, as previously detailed, we aimed to confirm the DASH fit to a one-factor model. This confirmation allowed us to obtain a single unidimensional questionnaire with a single total score, which is more practical than more subtest scores in the clinical context, considering, however, that the DASH explores not only motor function (items 1 to 21), but also pain, the overall impact on daily life, sleep and self-confidence (items 22 to 30). Besides, even in other populations, like humeral shaft fracture [77], hand and wrist injuries [78], and rheumatoid arthritis [79], DASH was proven to be unidimensional, and its total score can be used as a unique questionnaire. Finally, CFA can be used as a technique to preliminarily explore the dimensionality of the instrument, to obtain a sufficiently unidimensional instrument to then be subjected to Rasch analysis [80]. Indeed, Rasch analysis is a confirmatory analytical technique for validating a scale that is supposed to be unidimensional [80], therefore, the unidimensionality of different scales was first addressed with classical psychometric techniques [63, 81] and subsequently through Rasch analysis [82, 83].

Reliability analyses reported an excellent internal consistency of the total score as demonstrated by the high α (= 0.951) and good ITC value of 0.389 (cut-off > 0.200); this value is in line with the ones reported in other previous versions of the DASH [70, 84, 85].

Considering the internal consistency of the items, all items contribute to the generation of the total score, except three items (item#2, item#20, item#21) with low ITC. Regarding item#2, authors could speculate that writing is often performed with the dominant hand. However, as shown by demographic data, FS mainly affects the non-dominant hand, which could justify the low ITC value. Regarding item#20 and item#21, such specific activities (i.e., managing transportation needs and sexual activities) probably included skills involving other body parts out of the shoulder. Various strategies could be adopted as compensation for the impaired shoulder function. This could be a further justification for the unsatisfied cut-off.

The six missing answers to item#21 (*n* = 6/124, 4.84%) could be due to various reasons, such as misunderstanding the question, accidental oversight, or an individual’s reluctance to answer a particular query, as seemed that in such cultures and for some particular population (i.e. elderly people) this question remained a taboo. Notably, this feature is not uncommon as many studies [38, 69, 72, 86–91] reported missing answers to item#21, and such item has been consistently the most frequently omitted one. As reported in Cheng et al. 2008 [92], the prevalence in the literature of frequent non-response to item#21 may be attributed to the difficulties that subjects encounter in assessing aspects of their sexual life in contact with the examiner, or could be linked to cultural [71, 73, 93] and socio-religious factors [94], as discussing one’s sexual habits publicly could be deemed socially unacceptable.

The excellent test-retest reliability of this instrument is underlined by the data, as evidenced by an ICC value equal to 0.999 in the specific subset of the sample studied. This figure signifies a high level of consistency and agreement among the data points evaluated, which findings in existing literature have corroborated [71, 95]. Such test-retest reliability value serves to endorse the broad applicability of this instrument, making it suitable for use in clinical settings and research environments. This suggests that the tool can be relied upon to consistently measure the same construct over time, ensuring its utility and validity in various contexts. This high value in test-retest assessment could be due to the specific characteristics of the patients with FS. Indeed, subjects with FS with almost seven months from onset (as our sample) tend to remain stable longer than 5 days [4]; therefore, considering the characteristics of the FS, there’s no clinical progression within 5 days that test-retest reproducibility is excellent.

Evidence on reliability is also endorsed by measurement error assessment, which showed a SEM of 0.5 points and an MDC of 1.5 points. This low MDC value indicates that the DASH can detect even minimal changes in a patient’s condition, and this is particularly crucial in a clinical setting, where even small yet meaningful improvements can positively impact the patient’s life. We found a fairly low value of MDC, in contrast to other results from previous articles. In adults with musculoskeletal upper extremity problems, various MDC values have been found ranging from about 10 [96, 97] to over 12 points [98, 99]. It is necessary to point out that the MDC calculation considers the SEM; to calculate the latter, it is necessary to use the test-retest reliability coefficient (in our case, the ICC) and the SD of the DASH scores of the sample. In our sample, the ICC was very high (= 0.999) for the reasons explained above, and the SD of the DASH was quite small (= 17.2); indeed, our sample was quite homogeneous compared to previous studies [96–99]. Thus, the reduced variability of the DASH scores could be attributed to our small number of subjects used to evaluate test-retest reliability and to the fact that our patients were very similar to each other, as they were recruited from the same type of setting ( i.e., private rehabilitation centres) with the same disease, unlike the samples of previous studies that included several upper limbs disorders with different aetiologies [96–99]. Finally, comparing the MDC to the Minimally Clinical Importance Difference (MCID) helps clinicians to understand whether statistically significant changes are also clinically relevant, helping bridge the gap between statistical and clinical significance. Unfortunately, we could not investigate the MCID, so future studies focusing on this value would be of clinical usefulness.

The strength of the relationships observed in the data further bolsters the evidence for construct validity studied with classic psychometric techniques, suggesting that the scale is a valid tool for measuring the targeted construct in the studied population. In summary, these results about construct validity provide valuable insights into the robustness of the measurement instrument, affirming its ability to assess the intended construct effectively in most aspects.

Limited generalizability of the results arises from the highly specific sample used, and significant psychometric properties, such as construct validity assessed within the framework of Rasch Measurement Theory and responsiveness, were not investigated.

Furthermore, since this validation study specifically focused on Italian individuals with FS, our results should, in the future, be confirmed in other Italian people suffering from other shoulder-specific conditions (i.e., shoulder instability, rotator cuff tear patients). Lastly, the disagreement in the literature about “one” or “more than one” -factor model (i.e., unidimensional or multidimensional) evaluation of DASH needs further exploration to determine a consensus on the structural validity of the DASH questionnaire.

## Conclusions

In conclusion, this is the first study that assesses the DASH psychometric properties (i.e., reliability and construct validity) in a sample of subjects suffering from FS and helps to cover a gap in the literature, as no PROMs evaluated the validity of DASH in such a population.

DASH-I showed good psychometric properties in assessing a specific sample of subjects suffering from FS. It demonstrated high reliability and a satisfying one-factor structural validity for clinical and research use. Data suggest that this measurement tool, allowing patients to self-assess their treatment progress, will significantly enhance the overall evaluation modalities for individuals suffering from FS. Further studies are needed to explore other fundamental psychometric properties, such as construct validity with modern techniques (i.e., Rasch analysis) and responsiveness.

## Data Availability

The datasets used and/or analyzed during the current study available from the corresponding author on reasonable request.
